# POLR3-related leukodystrophy caused by biallelic *POLR3A* and *1C* pathogenic variants: a single-center experience

**DOI:** 10.3389/fneur.2024.1355484

**Published:** 2024-03-14

**Authors:** Jing Liu, Yue Niu, Jiong Qin, Zhixian Yang

**Affiliations:** ^1^Department of Pediatrics, Peking University People's Hospital, Beijing, China; ^2^Epilepsy Center, Peking University People's Hospital, Beijing, China

**Keywords:** POLR3-related leukodystrophy, *POLR3A*, *POLR1C*, Chinese population, mutation

## Abstract

**Objectives:**

This study aimed to investigate the clinical, radiological, and genetic features of POLR3-related leukodystrophy caused by mutations in *POLR3A* or *POLR1C*.

**Methods:**

Fourteen Chinese patients with POLR3-related leukodystrophy were enrolled in this cross-sectional observational study. The clinical manifestations, brain MRI and genetic tests of the patients were evaluated.

**Results:**

Thirteen patients had biallelic variants in *POLR3A* (92.9%), and one had biallelic variants in *POLR1C* (7.1%). The median age at disease onset was 9 months. A total of 85.7% of the patients presented with motor delay, abnormal gait, and intelligence disability in the first 2 years of life. Intellectual disability can be categorized based on its severity. It varied from mild (which involves difficulty concentrating) to very severe (with no smiling or laughing or never being able to speak since birth). Short stature was observed in all patients, and delayed dentition was observed in 64.3% of them. Furthermore, three out of 14 patients had myopia. Hypomyelination was invariably present in all patients, whereas myelination of the basal ganglia was preserved in only six out of 14 patients. All the mutations were compound heterozygous and included missense (*n* = 25), deletion (*n* = 1), and splice site variants (*n* = 2). A total of 78.6% of the patients with *POLR3A* were identified as carrying the c.1771-6C>G variant or the c.1771-7C>G variant.

**Conclusion:**

The phenotypic diversity of POLR3-HLD associated with pathogenic variants ranges from mild to very severe for neurological and non-neurological symptoms. Most patients presented symptoms in the first 2 years of life. The c.1771-6C>G or c.1771-7C>G variant is the most frequent mutation site in *POLR3A* in Chinese individuals.

## Highlights

- POLR3-HLD exhibits diverse neurological and non-neurological phenotypes.
- Most patients presented symptoms in the first 2 years of life.
- *POLR3A* hotspot mutations appear in the splice tract.

## 1 Introduction

POLR3-related hypomyelinating leukodystrophy (POLR3-HLD) refers to a rare autosomal recessive disease characterized by hypomyelination, which is often accompanied by dental abnormalities and hypogonadotropic hypogonadism (1–3). Brain MRI generally reveals diffuse hypomyelination associated with relative T2 hypointensity in the ventrolateral thalami, globi pallidi, optic radiations, and corticospinal tracts at the internal capsule level. Cerebellar atrophy and thinning of the corpus callosum are also common findings of this study (4–6).

The *POLR3A, POLR3B, POLR1C*, and *POLR3K* genes, which encode the RNA polymerase III (POLR3) complex, are relevant to POLR3-HLD (7, 8). *POLR3A* and *POLR3B* encode the largest subunits that form the catalytic core of RNA polymerase III (7, 9). *POLR1C* encodes a subunit of both RNA polymerase I and III, while *POLR3K* encodes a different subunit of RNA polymerase III (10). The proper function of the RNA polymerase III enzyme is crucial for the maintenance and development of myelin. A study in European populations revealed recessive mutations in *POLR3A* or *POLR3B* in fourteen patients; eight novel mutations were identified in *POLR3A*—six missense mutations, one nonsense mutation, and one frameshift mutation—and seven patients carried compound heterozygous mutations in *POLR3B* (11). In 2019, a total of 29 different variants in *POLR1C* were identified in 23 POLR3-HLD patients from 25 different centers worldwide. However, cases among the Asian population are scarce.

To date, several case reports on POLR3-HLD in the Chinese population have been published (12–15). In this study, we present a detailed description of the clinical, molecular, and radiological characteristics of 14 Chinese patients with mutation-proven POLR3-HLD.

## 2 Materials and methods

Fourteen children were included in this single-center cross-sectional retrospective study. The participants were recruited between January 2014 and March 2023 at Peking University People's Hospital based on clinical and radiological features by a POLR3-HLD diagnosis (16). All patients were identified by trio-based whole exome sequencing as having either POLR3A or POLR1C mutations. The demographic data, medical records, laboratory test results, and images of the patients were reviewed.

The study was approved by the ethics committee of Peking University People's Hospital and was conducted in accordance with the Declaration of Helsinki. The individuals or their parents in this manuscript provided written informed consent to publish the case details.

All available MRI data were analyzed for this study. MRI was performed with a GE750 3.0 T machine. The study typically included sagittal T1-weighted and axial T1-weighted, T2-weighted, T1 FLAIR, T2 FLAIR, and fluid-attenuated inversion recovery images.

Genomic DNA was extracted from the peripheral blood lymphocytes using standard protocols. Variant screening of *POLR3A* (NM_007055.4) and *POLR1C* (NM_203290.4) was performed using trio-based whole-exome sequencing and copy number variation (CNV) analysis by the Beijing Chigene Translational Medical Research Center Co. Ltd. Variants segregated within a family in an autosomal recessive or X-linked manner were ruled out if the max allele frequency (ExAC or gnomAD) was >0.001. *De novo* variants were ruled out if the max allele frequency (ExAC or gnomAD) was >0.0001. For candidate genes, variants were predicted by MutationTaster (http://www.mutationtaster.org/), Polyphen-2 (http://genetics.bwh.harvard.edu/pph2/), and SIFT (http://sift.jcvi.org/). Sanger sequencing with parental DNA samples were processed to verify whether the variants were inherited from parents or arose de novo. The pathogenicity of variants was interpreted according to the American College of Medical Genetics (ACMG) guidelines. Only pathogenic and likely pathogenic variants were considered disease-causing variants. Variants were described based on the reference sequence GRCh37 (NM_007055.4 for *POLR3A*, NM_018082.6 for *POLR3B*, and NM_203290.4 for *POLR1C*). Compliance with the Human Genome Variation Society (HGVS) nomenclature was verified using a variant validator (https://varnomen.hgvs.org/).

The incidence of myopia in this study and that was reported in the previous literature was compared using the chi-squared test. The statistical analysis was performed at a significance level of 0.05 using SPSS software, version 23.0.

## 3 Results

Among the 14 patients, seven were males (50%) and seven were females (50%). There were no consanguineous families in this study. The median age at disease onset was 9 months (ranging from 2 months to 9 years). The median current age was 5 years (ranging from 12 months to 17 years). The demographic and clinical characteristics of the patients are summarized in Table 1.

**Table 1:** The clinical characteristics of 14 patients with POLR3-HLD caused by biallelic *POLR3A* and *1C* pathogenic.

**Patient**	**Sex**	**Current age**	**Siblings**	**Age at onset**	**Symptoms at onset **	**Dysphagia**	**Axial hypotonia**	**Ataxia**	**Nystagmus**	**Pyramidal signs**	**Myopia**	**Intelligence delay**	**Short stature**	**Retarded dentition**	**Microcephaly**	**Seizure**	**Death**
1	Male	6 years	A sister, unaffected	1 year	Global developmental delay	–	+	–	–	+	–	+	+	+	–	–	–
2	Male	7 years	No	6 months	Global developmental delay	–	+	_+_	_−_	+	–	+	+	+	–	+	–
3	Female	5 years	Pt4	6 months	Global developmental delay	–	+	+	–	–	–	+	+	+	–	–	–
4	Male	4 years	Pt3	5 months	Motor delay	–	+	–	–	–	–	+	+	+	–	+	–
5	Male	2 years	No	8 months	Motor delay	+	+	–	+	–	–	+	+	–	+	–	–
6	Female	9 years	No	1 year 6 months	Abnormal gait, prone to falling	–	+	+	–	+	–	+	+	–	–	–	–
7	Male	17 years	Pt8	9 years	Abnormal gait, prone to falling	+	+	+	+	+	–	–	+	–	–	–	–
8	Female	14 years	Pt7	9 years	Abnormal gait, prone to falling	–	+	+	–	+	+	+	+	–	–	–	–
9	Female	7 years	Pt10	2 months	No smiling, absent visual contact, Low algesthes	+	+	+	–	+	–	+	+	+	+	–	•+ •at 7 years
10	Female	12 months	Pt9	2 months	No smiling, No crying, absent visual contact, Low algesthes	+	+	+	–	–	–	+	+	+	+	–	–
11	Female	15 months	No	2 months	No smiling, Global developmental delay	+	+	–	–	–	–	+	+	+	–	–	–
12	Male	20 months	No	3 months	Global developmental delay	+	+	–	–	–	+	+	+	+	+	–	–
13	Male	3 years	No	1 year 7 months	Abnormal gait, walk unsteadily	–	+	+	–	–	–	–	+	–	–	–	–
14	Female	5 years	No	1 year 9 months	Abnormal gait	–	+	+	+	+	+	+	+	+	–	–	–

y, year; m, month; P, patient.

### 3.1 Neurologic and non-neurologic manifestations

Within the first 2 years of life, 11 out of 14 (78.6%) patients presented symptoms. For most patients, the initial symptoms included motor delay, abnormal gait, and intellectual delay. Furthermore, 78.6% of the patients (11/14) were never been able to walk independently, and their ages ranged from 12 months to 9 years. Most patients exhibited severe intention tremors and ataxia, while pyramidal signs usually develop slowly in older patients. Out of 14 patients, six (41%) had dysphagia, and five of them required a gastrostomy tube. A total of 21.4% of the patients (3/14; two with *POLR3A* and one with *POLR1C*) had myopia. The frequency of myopia in our cohort was lower than that in previously reported patients with *POLR3A* or *POLR3B* variants (87%) (1) (*P* < 0.001). Most patients presented with intellectual disability that ranged from mild (only with difficulty concentrating) to very severe (with no smiling or laughing or never being able to speak since birth). Six (42.9%) patients experienced neurological deterioration with infections. One patient died due to disease progression at 7 years of age. Two of the children had epileptic seizures during the course of the disease, both of which were epileptic spasms. One patient started experiencing seizures at 7 years of age, which were later reduced with the use of topiramate. The other patient experienced only one seizure at the age of 4 years and was not treated with anti-seizure medications.

Delayed dentition with an abnormal order of deciduous tooth eruption was found in nine out of 14 patients (64.3%). Their median time to dental eruption was 13 months and the median time to full dentition was 5.2 years. The abnormal order of deciduous tooth is manifested by the intermittent and abnormal eruption of molar teeth during the eruption of incisors. Twelve patients were between the age of 12 months and 9 years and had not yet developed secondary sexual characteristics. However, there were no signs of arrested puberty or absence of early pubertal changes observed in the only two patients who reached puberty. They were not tested for values of sex hormones.

All 14 patients had short stature. The heights of 10 children were below the 3rd percentile of the same age, three were between the 3rd and 10th percentiles, and one was in the 25th percentile. The height of five patients never increased after the disease started to develop. However, none of the patients were treated with growth hormones.

### 3.2 MRI findings

**Figure 1:**
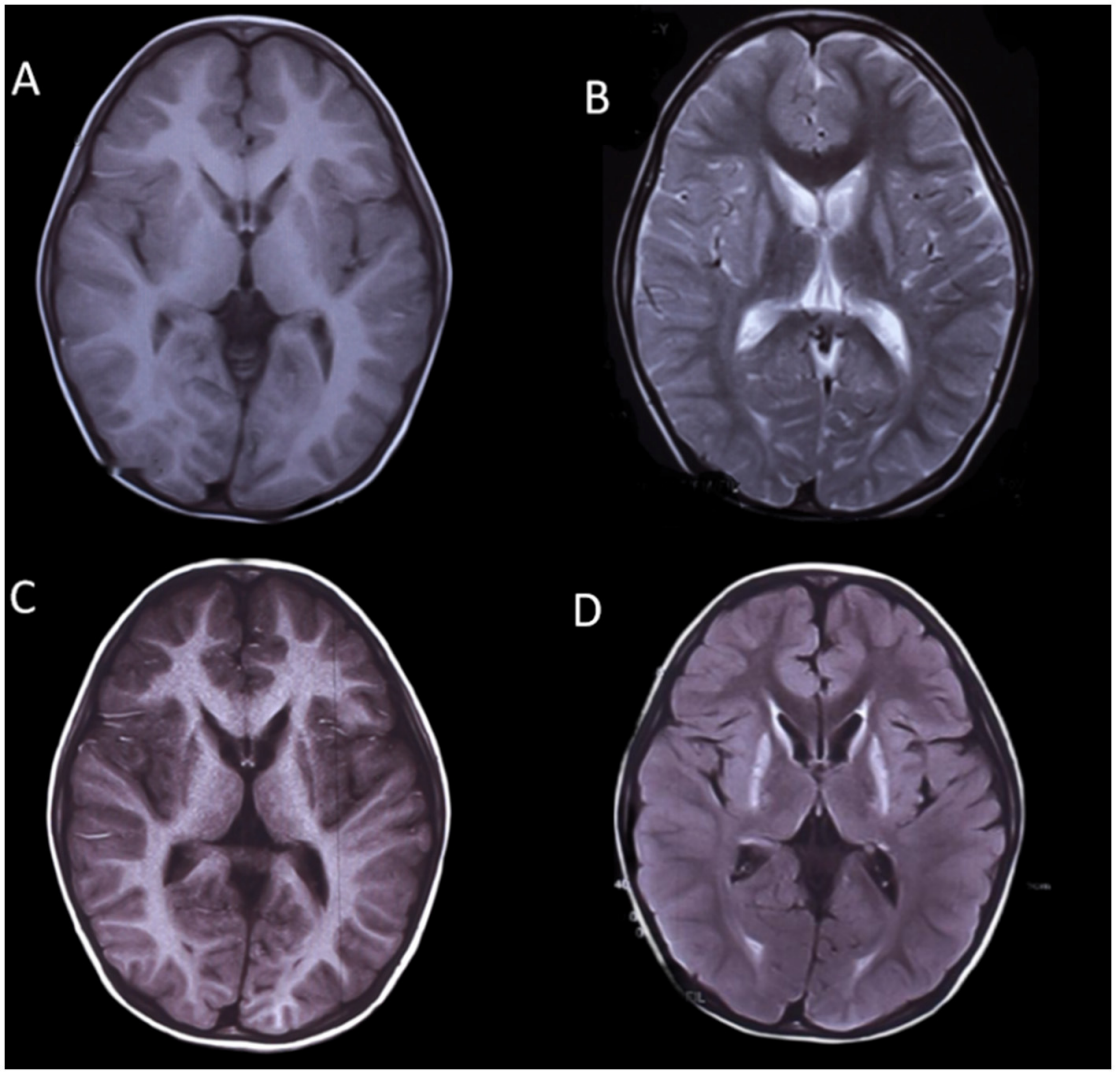
MRI images of a boy with POLR3-HLD (Patient 2), **(A, B)** at 4 years of age and **(C, D)** at 7 years of age. The MRI image shows T1-hyperintense and T2-hyperintense of bilateral caudate nucleus and lentiform nucleus.

**Figure 2:**
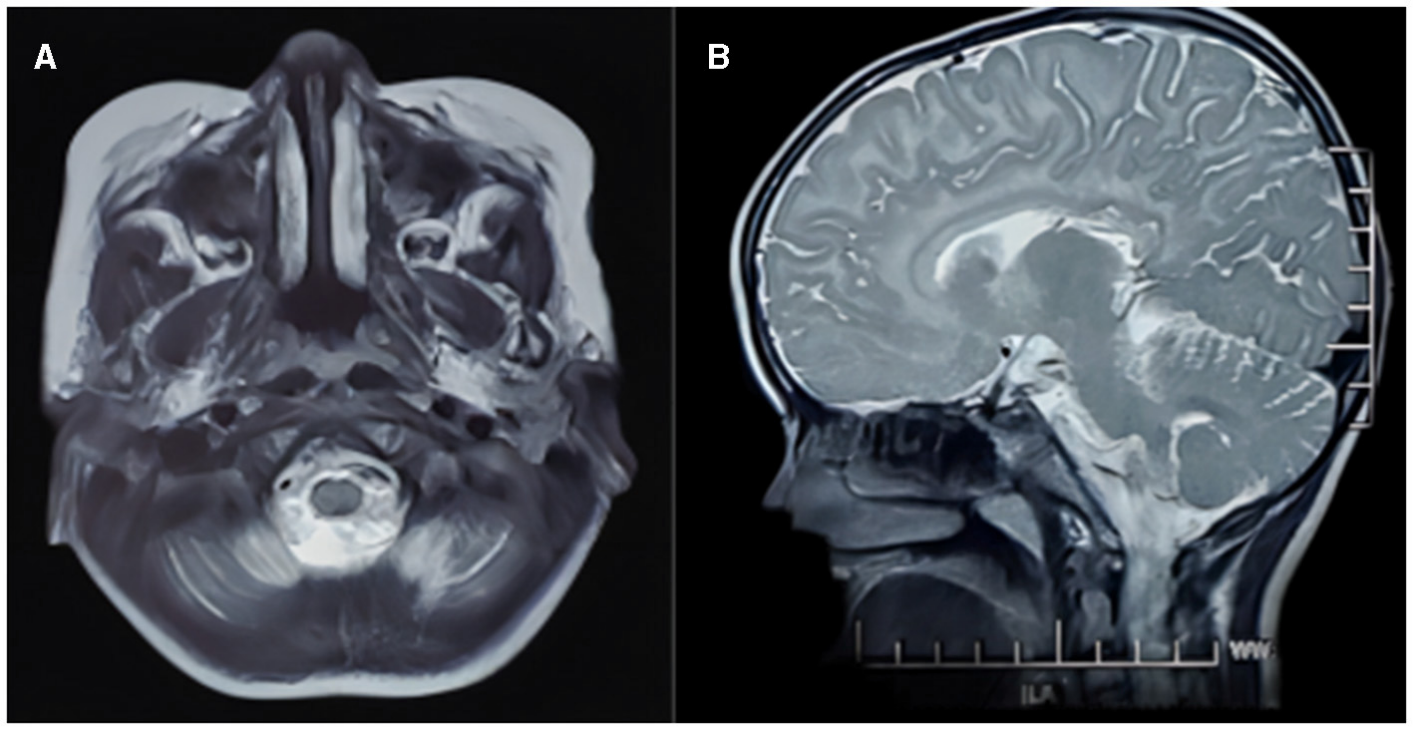
MRI images of a girl with POLR3-HLD (Patient 14), **(A, B)** at 5 years of age. The MRI image shows patchy T2WI hypersignal in the dentate nucleus of the cerebellum and thin corpus callosum with T2WI hypersignal.

Brain MRI images were available for all participants. In eight patients, abnormal MRI signals were first found between 1 and 2 years of age, and in two patients, they were between 2 and 4 years of age. Hypomyelination was invariably present in all patients (14/14; 100%). Preserved myelination of the basal ganglia was seen in six out of 14 (42.9%) participants (patients 1, 2, 3, 4, 6, and 9; Figure 1), and the pyramidal tracts in the posterior limbs of the internal capsules were seen in two out of 14 (14.3%) participants (patients 7 and 8). The MRI images of patient 2 showed T1-hyperintense and T2-hyperintense of bilateral caudate nucleus and lentiform nucleus at 4 and 7 years of age, respectively (Figure 1). Caudate nucleus atrophy was observed in patients 3 and 4. T2 hyperintensity in the dentate nucleus of the cerebellum was present in two patients (14.3%). Thinning of the corpus callosum with T2WI hypersignal was observed in one out of 14 (7.1%) patients (patient 14), and the MRI image also showed patchy T2WI hypersignal in the dentate nucleus of the cerebellum in patient 14 (Figure 2).

### 3.3 Molecular findings

All 14 patients had a confirmed diagnosis of POLR3-HLD based on their clinical and radiological features in addition to the molecularly confirmed presence of likely pathogenic or pathogenic variants in *POLR3A* or *POLR1C*. Thirteen patients had biallelic variants in *POLR3A* (92.9%), and one had biallelic variants in *POLR1C* (7.1%; Table 2). All mutations were compound heterozygous, including missense variants, deletion variants, and splice site variants. The most common variants were c.1771-7C>G, identified in six individuals from five unrelated families (patients 2, 5, 9, 10, 11, and 12), and c.1771-6C>G, identified in five individuals from three unrelated families (patients 1, 3, 4, 7, and 8). The combination of c.1771-7C>G and c.3429+51A>G was present in two siblings, and they presented symptoms in early infancy and experienced an earlier onset age of 2 months and a more severe phenotype, with only achieving some head balance but no laughing or smiling. Among the mutations identified, 12 were novel and were not reported in public databases. The segregation analysis of this family revealed that each of the parents carried one variant.

**Table 2:** Genetic characteristics of 14 POLR3-HLD patients.

**Patient**	**Family**	**Gene**	**Nucleotide change**	**amino acid change**	**Inheritance**	**SIFT**	**Polyphen**	**Mutation taster**	**Allele frequency in updated gnom AD (v2.1.1 and v3.1.1)**	**Reported in the literature or databases**
1	I	POLR3A (NM_007055.3)	c.1771-6C>G	–	Heterozygous father	–	–	–	0	Yes
		POLR3A	c.733G>A	p.(G245R)	Heterozygous mother	Probably damaging	Probably damaging	Prediction disease-causing	0.000007072	No
2	II	POLR3A	c.2044C>T	p.(R682W)	Heterozygous father	Probably damaging	Probably damaging	Prediction disease-causing	0	No
		POLR3A	c.1771-7C>G	–	Heterozygous mother	–	–	–	0	Yes
3	III	POLR3A	c.1771-6C>G	–	Heterozygous father	–	–	–	0	Yes
		POLR3A	c.1980G>C	p.(K660N)	Heterozygous mother	Probably damaging	Probably damaging	Prediction disease-causing	0.00003186	No
4	III	POLR3A	c.1771-6C>G	–	Heterozygous father	–	–	–	–	Yes
		POLR3A	c.1980G>C	p.(K660N)	Heterozygous mother	Probably damaging	Probably damaging	Prediction disease-causing	0.00003186	No
5	IV	POLR3A	c.1572+5G>A	–	Heterozygous father	–	–	–	0	No
		POLR3A	c.1771-7C>G	–	Heterozygous mother	–	–	–	0	Yes
6	V	POLR3A	c.2564G>A	p.(R855Q)	Heterozygous father	Probably damaging	Probably damaging	Prediction disease-causing	0	No
		POLR3A	c.1781T>G	p.(L594R)	Heterozygous mother	Probably damaging	Probably damaging	Prediction disease-causing	0	No
7	VI	POLR3A	c.1787C>T	p.(T596M)	Heterozygous mother	Probably damaging	Probably damaging	Prediction disease-causing	0	No
		POLR3A	c.1771-6C>G	–	Heterozygous father	–	–	–	0	Yes
8	VI	POLR3A	c.1787C>T	p.(T596M)	Heterozygous mother	Probably damaging	Probably damaging	Prediction disease-causing	0	No
		POLR3A	c.1771-6C>G	–	Heterozygous father	–	–	–	0	Yes
9	VII	POLR3A	c.1771-7C>G	–	Heterozygous father	–	–	–	0	Yes
		POLR3A	c.3429+51A>G	–	Heterozygous mother	–	–	–	0	No
10	VII	POLR3A	c.1771-7C>G	–	Heterozygous father	–	–	–	0	Yes
		POLR3A	c.3429+51A>G		Heterozygous mother	–	–	–	0	No
11	VIII	POLR3A	c.1771-7C>G	–	Heterozygous mother	–	–	–	0	Yes
		POLR3A	c.775C>T	p.(P259S)	Heterozygous father	Probably damaging	Probably damaging	Prediction disease-causing	0	No
12	IX	POLR3A	c.2273_2274delTG	p.(V758Dfs^*^3)	Heterozygous father	–	–	–	0	No
		POLR3A	c.1771-7C>G	–	Heterozygous mother	–	–	–	0	Yes
13	X	POLR3A	c.1787C>T	p.(T596M)	Heterozygous father	Probably damaging	Probably damaging	Prediction disease-causing	0	No
		POLR3A	c.1727A>G	p.(D576G)	Heterozygous mother	Probably damaging	Probably damaging	Prediction disease-causing	0	No
14	XI	POLR1C (M_203290.4)	c.424C>T	p.(L142F)	Heterozygous father	Probably damaging	Probably damaging	Prediction disease-causing	0	Yes
		POLR1C	c.53A>T	p.(E18V)	Heterozygous mother	Probably damaging	Probably damaging	Prediction disease-causing	0	No

## 4 Discussion

In this study, we analyzed the clinical and genetic features of 14 Chinese children with POLR3-HLD. Notably, POLR3-HLD has mostly been reported in European populations (1, 17). Occasionally, cases have been reported in the Chinese population (13, 14). This study presents the detailed clinical and molecular characteristics of POLR3-HLD in a Chinese population. We identified the most frequent mutation site in *POLR3A* and identified 12 novel variants not previously reported in the Chinese population.

In general, patients with *POLR3A* and *1C* variants are more severely affected than patients with *POLR3B* mutations, with faster regression and shorter life expectancy (1, 18). POLR3-HLD with *POLR1C* variants has been relatively less reported. Previous studies have shown that the *POLR1C* variant is more severe than the previously reported *POLR3A* variant (18). However, in our study, the symptoms of the patients with *POLR3A* variants seemed to be more severe than those with *POLR1C* variants. It is possible that our results were influenced by the small sample size of patients with *POLR1C* variants. Our findings suggest that POLR3-HLD is a spectrum of clinical features of varying severity, including declining motor function, increasing ataxia, and mild to moderate intellectual disability. We discovered that patients with earlier symptom onset usually had a more severe phenotype and did not achieve ambulation. Neurological impairment started in patients 9 and 10 in the early infantile period, with motor ability and cognitive impairment; patients achieved only some head balance but no laughter or smiling, and patient 9 was unable to survive. Ataxia is prevalent in almost two-thirds of the patients. Nystagmus was observed in a few patients. Two patients were observed to have seizures. This finding is consistent with previous studies (5, 19, 20). Seizures were usually infrequent in POLR3-HLD patients. Wolf et al. reported seizures in 19 out of 99 (19%) patients, which were usually well controlled with medication. However, the type of the seizure was not reported in detail. Harting et al. reported seizures in one out of nine (11.1%) patients aged 15 months with myoclonic jerks. Epileptic spasms have not been reported previously.

Our study showed that the endocrine abnormalities were found to vary significantly. Notably, all patients in this study presented with short stature, exceeding a greater proportion reported in previous research studies (1). Delayed dentition was observed in nearly two-thirds of the patients. However, there was no arrested puberty or absence of early pubertal changes observed in the only two patients who reached puberty. Myopia was observed in only three patients. The frequency of myopia in our cohort was lower than that in previously reported patients with *POLR3A* or *POLR3B* variants (1). This could be partly due to population differences or the fact that our patients were young since myopia is known to progress in patients with POLR3-HLD over time. Therefore, it is important to screen patients for short stature, dental abnormalities, and myopia to provide multidisciplinary team care (11, 21–23). On the other hand, POLR3-HLD should be suspected in individuals with these neurological and non-neurological manifestations mentioned above.

Diagnosing POLR3-HLD in the absence of dental and endocrine abnormalities is challenging. The brain MRI pattern is an important diagnostic indicator. In POLR3-HLD patients, brain MRI generally reveals diffuse hypomyelination with relative myelin preservation of specific brain structures, including the dentate nuclei, anterolateral nuclei of the thalami, globi pallidi, pyramidal tracts in the posterior limbs of the internal capsules, and optic radiations. Cerebellar atrophy and hypoplasia of the corpus callosum are variably observed, as in our patients. The incidence of thinning of the corpus callosum is lower compared to the previous study (18). Although diffuse hypomyelination was observed in most of our patients, it is not a mandatory diagnostic imaging feature, as shown in the literature (24, 25).

In our cohort, most patients with *POLR3A* mutations were identified as carrying the c.1771-6C>G variant or the c.1771-7C>G variant in combination with another variant. The c.1771-6C>G or c.1771-7C>G variant is the most frequent mutation site in *POLR3A* in Chinese individuals. The recurrence of these variants among Chinese patients may alternatively correspond to ancient founder effects, increasing the allele frequencies in such a specific population. In addition, we found that basal ganglia involvement was more likely to be detected via brain MRI in patients harboring any of these two variants. However, basal ganglia involvement were not observed for some patients (patients 5, 10, 11, and 12), possibly because they were young and had not yet progressed to this stage. The c.1771-7C>G variant has been shown to result in two aberrant transcripts in addition to the normal cDNA, interpreted as activating a leaky splice site with both wildtype and aberrant transcripts (26). The c.1771-6C>G variant has been shown to skip exon 14 and cause premature termination of some of the transcripts, with the shorter transcript being subject to nonsense-mediated decay (27). Furthermore, in this study, *POLR3A* was more common than *POLR1C*, possibly due to the higher incidence of *POLR3A* and the small number of enrolled patients.

In summary, this study comprehensively characterized the clinical, brain magnetic resonance imaging, and genetic features of POLR3-associated leukodystrophy caused by autosomal recessive inheritance of *POLR3A* and *1C*. We also found diverse disease spectrum phenotypes ranging from mild to severe associated with pathogenic variants. Moreover, we reported new mutation sites in *POLR3A* and *POLR1C* for the first time and identified the most frequent mutation site in *POLR3A* in Chinese individuals. This study will aid in understanding POLR3-related leukodystrophy and will promote further analysis of phenotype–genotype correlations of this disease in the future. This study has several limitations that must be considered. Specifically, the number of cases is relatively small, and there are some unidentified variants. In the future, multicenter studies with POLR3-associated leukodystrophy could be conducted, expanding the phenotype in rare diseases, and the pathogenicity might require further functional validation.

## Data availability statement

The datasets presented in this article are not readily available because of ethical and privacy restrictions. Requests to access the datasets should be directed to the corresponding authors.

## Ethics statement

The studies involving humans were approved by The Ethical Committee of Peking University People's Hospital. The studies were conducted in accordance with the local legislation and institutional requirements. Written informed consent for participation in this study was provided by the participants' legal guardians/next of kin.

## Author contributions

JL: Writing—original draft, Data curation, Investigation. YN: Methodology, Writing—original draft. JQ: Writing—review & editing. ZY: Supervision, Writing—review & editing.
